# Single-cell epigenomics: powerful new methods for understanding gene regulation and cell identity

**DOI:** 10.1186/s13059-016-0944-x

**Published:** 2016-04-18

**Authors:** Stephen J. Clark, Heather J. Lee, Sébastien A. Smallwood, Gavin Kelsey, Wolf Reik

**Affiliations:** Epigenetics Programme, Babraham Institute, Cambridge, CB22 3AT UK; Wellcome Trust Sanger Institute, Hinxton, Cambridge, CB10 1SA UK; Friedrich Miescher Institute for Biomedical Research, Maulbeerstrasse 66, CH 4058 Basel, Switzerland; Centre for Trophoblast Research, University of Cambridge, Cambridge, CB2 3EG UK; Department of Physiology, Development and Neuroscience, University of Cambridge, Cambridge, CB2 3EG UK

## Abstract

Emerging single-cell epigenomic methods are being developed with the exciting potential to transform our knowledge of gene regulation. Here we review available techniques and future possibilities, arguing that the full potential of single-cell epigenetic studies will be realized through parallel profiling of genomic, transcriptional, and epigenetic information.

## Introduction

Epigenetics involves the study of regulatory systems that enable heritable changes in gene expression within genotypically identical cells. This includes chemical modifications to DNA and the associated histone proteins, as well as changes in DNA accessibility and chromatin conformation [1]. Until recently, our understanding of these epigenetic modifications has depended entirely upon correlations between bulk measurements in populations of cells. These studies have classified epigenetic marks as being associated with active or repressed transcriptional states, but such generalizations often conceal a more complex relationship between the epigenome and gene expression.

Arguably, and as for many biological questions, investigation of epigenetic regulation in general is most usefully studied at the single-cell level, where intercellular differences can be observed leading to a more refined understanding compared with bulk analysis [2]. Additionally, the development of single-cell technologies is key to investigating the profound remodeling of the epigenome during the early stages of embryonic development, including in human samples where cell numbers are very limited and where epigenetic heterogeneity may be most pronounced.

**Fig. 1:**
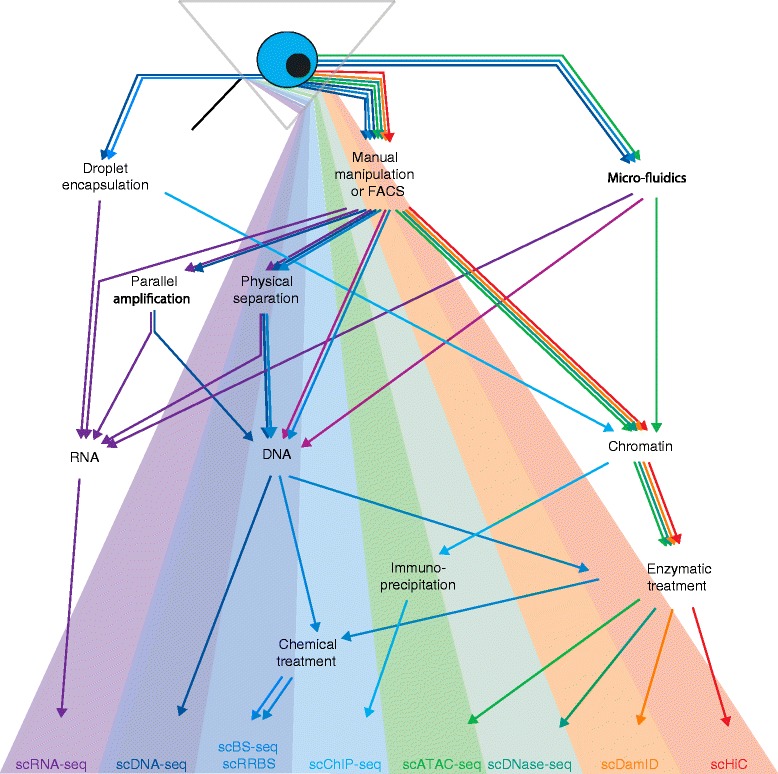
Epigenomics and the spectrum of single-cell sequencing technologies. The diagram outlines the single-cell sequencing technologies currently available. A single cell is first isolated by means of droplet encapsulation, manual manipulation, fluorescence-activated cell sorting (*FACS*) or microfluidic processing. The first examples of single-cell multi-omic technologies have used parallel amplification or physical separation to measure gene expression (*scRNA-seq*) and DNA sequence (*scDNA-seq*) from the same cell. Note that single-cell bisulfite conversion followed by sequencing (*scBS-seq*) is not compatible with parallel amplification of RNA and DNA, as DNA methylation is not conserved during in vitro amplification. Single-cell epigenomics approaches utilize chemical treatment of DNA (bisulfite conversion), immunoprecipitation or enzymatic digest (e.g., by DNaseI) to study DNA modifications (*scBS-seq* and *scRRBS*), histone modifications (*scChIP-seq*), DNA accessibility (*scATAC-seq*, *scDNase-seq*), chromatin conformation (*scDamID, scHiC*)

High-throughput sequencing has revolutionized the field of epigenetics with methods for genome-wide mapping of DNA methylation, histone modifications, chromatin accessibility, and chromosome conformation (Table 1). Initially, the input requirements for these methods meant that samples containing hundreds of thousands or millions of cells were required; but in the last couple of years this has changed with numerous epigenetic features now assayable at the single-cell level (Fig. 1). Combined single-cell methods are also emerging that allow analyses of epigenetic–transcriptional correlations thereby enabling detailed investigations of how epigenetic states are associated with phenotype.

**Table 1:** Survey of current and emerging single-cell epigenetics techniques

Technique	Epigenomic feature	Method	Approach	Single cell
Cytosine modification	5mC	BS-seq	Bisulfite converts C but not 5mC (or 5hmC) to U so only methylated sites are sequenced as “C”	Yes [6–8]
	5mC	MeDIP-seq	Immunoprecipitation of 5mC DNA followed by sequencing	Not currently possible
	5mC	Methyl-seq	Restriction enzyme specific for 5mC followed by sequencing	Possible
	5hmC	oxBS-seq	5hmC is oxidized to 5caC so that only 5mC survives bisulfite conversion. Readout is pure 5mC and subtraction from BS-seq determines 5hmC	Not possible for measuring 5hmC due to the need for subtraction
	5hmC	TAB-seq	Maps 5hmC by enzymatic oxidation prior to bisulfite treatment: only 5hmC survives conversion	Possible
	5hmC	hMeDIP-seq	Immunoprecipitation of 5hmC DNA followed by sequencing	Not currently possible
	5hmC	Aba-seq	Restriction enzyme specific for 5hmC	Possible
Protein–DNA interaction	Histone modification	ChIP-seq	Immunoprecipitation of DNA bound to a specific histone variant or transcription factor	Yes [17]
	Transcription factor binding	DamID	Cells are transfected with a fusion of a transcription factor gene and Dam protein which methylates adenine residues in proximity to the binding site. 6 mA specific restriction digest is used to map	Yes for nuclear lamina interactions [18]
Chromatin structure	Nucleosome positioning	MNase-seq	Microcococal nuclease digestion of chromatin and sequencing of the product which are regions protected by nucleosomes	Possible
	Nucleosome positioning	NOME-seq	GpC methylation of DNA not protected by nucleosomes followed by BS-seq	Possible
	DNA accessibility	DNase-seq	DNaseI digestion of open chromatin into small fragments suitable for library preparation and sequencing	Yes [23]
	DNA accessibility	FAIRE-seq	Chromatin is crosslinked, sonicated, and then purified by phenol–chloroform extraction. The aqueous layer contains only DNA not associated with protein	Not currently possible
	DNA accessibility	ATAC-seq	Tn5 transposase enzyme fragments and attaches adapters to open chromatin	Yes [21, 22]
Three-dimensional organization	Chromosome conformation	HiC	DNA is crosslinked, then restriction digested to fragment before ligation and reversal of the crosslinks. Resulting fragments are hybrids from separate genomic locations that were in close proximity in three-dimensional space. Paired-end sequencing is used to link the two regions	Yes [29]

*C* cytosine, *5caC* 5-carboxylcytosine, *5hmC* hydroxymethylcytosine, *5mC* methylcytosine, *U* uracil

In this article, we review current and emerging methods for mapping epigenetic marks in single cells and the challenges these methods present. We subsequently discuss applications of these technologies to the study of development and disease.

## Single-cell methodologies and future technological developments

### Cytosine methylation and other DNA modifications

DNA methylation of cytosine (5mC) residues can be mapped genome-wide using several methods such as methylation-specific restriction enzymes [3], affinity purification [4], or by using bisulfite conversion followed by sequencing (BS-seq) [5]. The latter is considered the gold-standard method as it allows single base resolution and absolute quantification of DNA methylation levels. While investigation of DNA methylation at the single-cell level was motivated by important biological questions, until recently it was unfeasible due to the large amount of DNA degradation caused by the bisulfite conversion, which was traditionally performed after preparing adapter-tagged libraries.

The first single-cell method for measuring genome-wide 5mC used a reduced representation bisulfite sequencing (scRBBS) approach based on enrichment of CpG dense regions (such as CpG islands) via restriction digestion, and it allows the measurement of approximately 10 % of CpG sites [6]. scRRBS is powerful because it allows assessment of a large fraction of promoters with relatively low sequencing costs, but its limitation is poor coverage of many important regulatory regions such as enhancers.

To develop true whole-genome single-cell approaches [7, 8] technological developments have been based on a post-bisulfite adapter-tagging (PBAT) approach in which bisulfite conversion is performed before library preparation so that DNA degradation does not destroy adaptor-tagged fragments [9]. As a result, methylation in up to 50 % of the CpG sites in a single cell can now be measured and this has allowed, for example, the detection of high variability between single cells in distal enhancer methylation (not usually captured by scRRBS) in mouse embryonic stem cells (ESCs) [7].

Building on this method has allowed BS-seq and RNA-seq in parallel from the same single cell (scM&T-seq) [10]. This was made possible by way of a method for physical separation of poly-A mRNA from DNA (genome and transcriptome sequencing or G&T-seq [11]), and this now allows intricate investigations of links between epigenetic and transcriptional heterogeneity within a particular cell and tissue type.

Hydroxymethylated cytosine (5hmC) is also thought to have a role in epigenetic gene regulation and has been analyzed in bulk samples using modified bisulfite sequencing methods [12, 13], 5hmC-specific restriction enzymes [14], or immunoprecipitation [15]. Of the currently established methods, TET-assisted bisulfite sequencing (TAB-seq) [12] and Aba-seq [14] could potentially be adapted to single cells. In TAB-seq, 5hmC is first enzymatically glucosylated in order to prevent its recognition by TET1, which is then used to oxidize 5mC to 5-formylcytosine and 5-carboxylcytosine which, along with unmodified cytosines, are sensitive to bisulfite conversion. These initial enzymatic steps could be performed in a single-tube reaction immediately before processing by single-cell BS-seq (scBS-seq). In Aba-seq, 5hmc is glucosylated prior to digestion with AbaSI, an enzyme that recognizes 5-glucosylhydroxymethylcytosine, and then prepared for sequencing by adapter ligation. Importantly, both of these techniques would be compatible with DNA purified using G&T-seq [11] thus allowing parallel measurements of 5hmC and poly-A RNA within the same single cell.

### Histone modifications and transcription factor binding

Histones can carry a diversity of covalent modifications that are associated with different genomic features and transcriptional states [16]. Mapping of histone marks is typically carried out using chromatin immunoprecipitation followed by sequencing (ChIP-seq). Performing ChIP-seq at the single-cell level is extremely challenging due to background noise caused by nonspecific antibody pull-down, which increases as the level of target antigen decreases. This was overcome recently by performing the immunoprecipitation step on chromatin from a pool of single cells that had already undergone micrococcal nuclease (MNase) digestion and barcoding, so that the pull-down is effectively performed on thousands of cells. This approach used a droplet-based microfluidics setup to process large numbers of cells in parallel [17], and because only a limited number of valid sequencing reads are obtained per single cell a large number of cells has to be sequenced in order to evaluate intercellular variability.

Protein–DNA interactions in single cells have been mapped using DamID, in which a cell line expresses low levels of a fusion protein of *Escherichia coli* deoxyadenosine methylase (Dam) and the protein under study. Dam methylates DNA on adenine residues adjacent to sites of protein binding. Methylated sites are then cut by the methylation-sensitive restriction enzyme DpnI, followed by ligation of sequencing adapters. This technique has been successfully employed to study interactions with the nuclear lamina in single cells [18]. Currently resolution is in the order of 100 kb, which to some extent limits its applications, but future optimizations could see improvements such that it could be used for mapping transcription factor binding sites in single cells. In addition, single-cell DamID could also support genome-wide analysis of histone modifications by using Dam fusion with specific histone readers or modifiers.

### Chromatin structure and chromosome organization

A raft of publications was seen in 2015 describing methods for mapping open chromatin in single cells. The first of these was based on the assay for transposase-accessible chromatin (ATAC-seq) which uses a Tn5 transposase enzyme to simultaneously fragment DNA and attach adapter sequences in a process called tagmentation [19]. Open chromatin regions can be defined by introducing the transposase into intact nuclei, where it acts on only DNA free of nucleosomes and transcription factors [20]. ATAC-seq was first adapted to single-cell resolution by employing a “combinatorial indexing” strategy in which the tagmentation is carried out on 96 pools of a few thousand nuclei, introducing a unique barcode to each pool. The 96 reactions are then pooled and split before a second barcode is introduced by polymerase chain reaction (PCR). The number of pools and cells per pool are optimized so that the probability that a particular barcode combination originates only from a single cell is kept sufficiently high [21]. In parallel a second single-cell ATAC-seq method has been described, which makes use of a commercially available microfluidics device to carry out the transposition reaction on individual cells [22]. This approach has resulted in a large increase in resolution compared with the combinatorial indexing method, mapping an average of 70,000 reads per cell compared with 3000, although throughput was substantially lower. Finally, investigation of open chromatin genomic regions has been achieved in single cells by employing a DNase-seq approach to map regions that are DNaseI hypersensitive. scDNase-seq provides an improved resolution of 300,000 mapped reads per single cell, albeit with a very low mapping efficiency (2 %) and even lower throughput [23]. Both of these methods could be combined with RNA-seq, either by way of physical separation [10] or parallel amplification [24].

In bulk samples, genome-wide nucleosome occupancy has been assayed by sequencing the products of MNase digestion [25] and by nucleosome occupancy and methylome sequencing (NOMe-seq) [26]. In NOMe-seq, a methyltransferase enzyme is used to methylate exposed GpC dinucleotides while DNA bound by nucleosomes is protected. Sequencing of the bisulfite-converted DNA can then be used to map nucleosome positions and this is particularly attractive for single-cell use since it will also give a readout of CpG methylation within the same single cell. Indeed nucleosome positioning has already been studied using locus-specific bisulfite PCR in the yeast *PHO5* gene, which revealed significant variability between cells that correlated with gene expression [27]. Single-cell nuclei prepared according to this method should be compatible with scBS-seq.

In addition to defining the linear chromatin organization of single cells, it is now possible to assess chromosome conformation at the single-cell level using a HiC-based method [28, 29]. Single-cell HiC is currently limited in its resolution but still allows description of the individual chromosome organization and compartmentalization, as well as interchromosomal interactions. This is a good example of how single-cell approaches can really provide cutting-edge tools, as regular HiC was traditionally performed on millions of cells resulting in an average of all chromosome organization within the cell population and hence some ambiguity in interpretation of the results.

### Advances in equipment to perform single-cells methods

Development of single-cell approaches is intimately linked to the development of physical equipment and devices. The first step in any single-cell analysis is the isolation and lysis of single cells from culture or dissociated tissue. This can be performed manually with a pipette and a microscope but such methods cannot realistically be scaled up for higher-throughput requirements. Fluorescence-activated cell sorting (FACS) can be used to isolate many thousands of single cells into microtiter plates in a short time with the additional benefit of being able to select cells based on a subset of fluorescent markers. Microfluidics systems have been developed, such as the C1 from Fluidigm, in which cells are trapped in chambers in which lysis and RNA-seq library preparation can subsequently be carried out. One advantage of this system is that captured cells are photographed in the system meaning that morphology of cells and presence of doublets can be assessed post hoc; however, these devices are currently only low-to-medium throughput, typically processing only 96 cells at one time. Recently, an innovative approach to generating single-cell libraries using microfluidics has emerged, which allows a significant increase in library preparation throughput compared with other methods. Cells are encapsulated within aqueous droplets in flowing oil, in which early stages of library preparation including cell-specific barcoding are performed, before being pooled for downstream reactions. In this way, thousands of cells are processed in parallel, with vastly reduced costs per cell and improved sensitivities compared with conventional tube-based methods. So far this approach has been applied to RNA-seq [30–32] and ChIP-seq [17] but in principle it could also be adapted to other methods such as BS-seq. Commercialization of droplet-sequencing technologies has already begun, meaning that these single-cell methodologies will be easily accessible and will be able to achieve their full potential.

In parallel we are witnessing a significant improvement in the field of single-molecule sequencing technologies with the potential to measure DNA modifications directly from native DNA and over tens of kilobases on the same molecule. This is particularly relevant since PCR amplification, bisulfite treatment, and other manipulations involved in library preparation can introduce technical artifacts, e.g., CG bias in BS-seq libraries. There are currently two single-molecule sequencing technologies on the market. The first of these, single-molecule real-time sequencing [33] as employed by the Pacific Biosciences RSII and Sequel machines works by real-time measurements of incorporating nucleotides and has been shown to discriminate cytosine from 5mC and 5hmC, although much effort is yet required to enable this analysis to be performed in a flexible way and routinely due to the subtle and context-specific effects of DNA modifications on incorporation kinetics [34]. The other technology, marketed by Oxford Nanopore, uses measurements of electrostatic charge as a DNA strand passes through a protein nanopore. While this technology is still in its infancy, in principle modified bases such as 5mC and derivatives could be detected [35]. These technologies currently require microgram quantities of DNA and therefore are not directly applicable to single cells; however, the use of cell-specific barcoding followed by pooling many thousands of single cells could allow analysis of individual cells.

### Quality control of single-cell epigenomic libraries

Quality control of sequencing data is crucial in order to avoid technical artifacts. This is especially true of single-cell sequencing, which is technically noisy due to low amounts of starting material, often resulting in variable capture efficiencies. The large number of amplification cycles that are needed often means that reagent contamination or sample cross-contamination is a very real problem and so sequencing of negative controls is recommended. Mapping efficiency or coverage cut-offs are also useful in order to eliminate cells that have performed much worse than the average. The use of spike-in controls may also be useful for some methods, for example to measure underconversion and overconversion by bisulfite. Another important consideration is the effect of cell dissociation on downstream analysis, since harsh enzymatic digestion of solid tissues is thought to influence single-cell transcriptomic studies [36]. Although epigenomic profiles are generally thought to be more stable than transcriptomes, the dissociation of cells should be performed as quickly and as mildly as possible to minimize the potential influence on single-cell libraries. Finally, batch effects can have a profound influence on single-cell datasets [37], so it is important to process samples in parallel wherever possible.

### Computational challenges to analyzing single-cell epigenomic data

The main computational challenges in single-cell data arise from the technical variability in the methods; this is due to low and variable capture efficiencies and biases introduced during PCR. This is a problem since it can be difficult to determine whether an observed difference is due to biological or technical reasons. These have been discussed in detail with respect to single-cell RNA-seq [38] where technical variability can be measured and normalized by use of synthetic spike-ins and unique molecular identifiers that are introduced during reverse transcription [39]. Single-cell epigenomic methods would likely benefit from similar strategies. Additionally, local correlations in epigenetic marks and correlations between epigenetic features and the underlying genetic sequence mean that missing information can be imputed in order to reduce the effect of low coverage. Such methods are in development and will be much improved when combined single-cell technologies become more sophisticated.

## Future applications of single-cell epigenomics

### Single-cell approaches to refine our understanding of epigenetic regulation

As mentioned above, epigenetic modifications have been characterized as transcriptionally repressive or activating based on correlations made in bulk cell populations. However, growing evidence has exposed the naivety of this assumption and revealed the great complexity of epigenetic regulation. For example, 5mC has long been considered to be a transcriptionally repressive mark since promoter methylation is negatively correlated with gene expression. However, in some cases DNA methylation of gene bodies has been positively correlated with transcription, demonstrating that the genomic context can influence the biological outcome [40]. Furthermore, global DNA hypomethylation seen in naive ESCs is not associated with widespread transcriptional activation [41, 42], demonstrating that the strength of regulatory links between DNA methylation and transcription can also vary depending on the developmental stage and cellular context. Since the discovery of 5hmC and other oxidized derivatives of 5mC, the situation has become even less clear, with inconsistent reports on the biological functions of these modifications [43–46].

Therefore, the use of single-cell approaches has the potential to refine our understanding of DNA modifications as regulatory epigenetic marks. The recent development of combined single-cell methods (e.g., scM&T-seq) will be invaluable to such studies [10]. In addition, the very low levels of 5hmC measured in bulk cell samples (e.g., less than 5 % of CpG sites in primed ESCs) indicate that only a few cells in that population have this modification at any particular cytosine residue. Therefore, parallel profiling of 5hmC and transcription will impact profoundly on our understanding of this epigenetic mark. In the future, it may even be possible to assay multiple epigenetic features (e.g., DNA methylation and chromatin accessibility) together with gene expression in the same single cell, leading to further refinements in our view of the epigenomic influence on the transcriptome.

According to the classical definition, epigenetic modifications must be heritable through cell divisions. While the mechanisms governing 5mC maintenance during DNA replication have been well described [47], the inheritance of other components of the epigenome is understood poorly. For example, the means by which histone modifications are conserved through DNA replication remain unclear [48]. This represents another application of single-cell approaches, in which one can imagine in vitro systems where mother and daughter cells can be sequenced to reveal the distribution of epigenetic marks between these two cells. When coupled with manipulations of epigenetic modulators (e.g., knockout models of histone-modifying enzymes), such an approach would allow the true nature of epigenetic propagation to be elucidated.

### Single-cell approaches to understand developmental processes and improve regenerative medicine

Single-cell transcriptional profiling has revealed population substructure in various developmental contexts [31, 32, 49–52]. In combination with lineage-tracing experiments, this information can be used to decipher the cellular hierarchy underlying complex tissues, giving unprecedented information on the molecular mechanisms governing differentiation processes. Epigenetic mechanisms are conventionally thought to restrict cell-fate decisions during development [53], so single-cell epigenomics studies will add valuable detail to these tissue hierarchies. It is also not excluded that in certain situations epigenetic information could be instructive for cell-fate decisions, and finely timed combined single-cell profiling techniques may provide insights into this important question.

Embryonic development involves global remodeling of the mammalian epigenome [38–40], including incorporation of maternal histones into the paternal genome following fertilization, and mechanisms leading to global DNA demethylation in both the preimplantation embryo and developing primordial germ cells. For this reason, many single-cell epigenomics techniques have been applied to embryonic development, taking mouse ESCs as a model system [6–8, 10, 29]. These studies have revealed intercellular epigenetic heterogeneity in cells poised for differentiation, which may have biological importance in lineage priming [54].

The near future will undoubtedly witness the application of single-cell epigenomic approaches in vivo. For example, mouse zygotes fertilized in vitro and embryos resulting from natural matings will be studied to understand epigenome dynamics during this critical stage of development. Due to the low cell numbers associated with these samples, FACS isolation of single cells is infeasible, so single cells will be manually picked after embryo dissociation. For the early stages of development, it should be possible to study every cell isolated from an embryo, while at later time points (E6.5 onwards) the increasing cell number may necessitate focused studies on specific cell lineages or on representative subpopulations of each lineage. A limitation to these studies will be the loss of spatial information upon embryo dissociation. Complementary studies including in vivo imaging of lineage-specific genes will be used to map cell types identified by single-cell sequencing back to the three-dimensional embryo [55]. By employing single-cell multi-omics, these studies will reveal the fundamental processes of cell-fate specification and establish an atlas of differentiation in which every tissue type can be traced back to its embryonic origins. This information will bring light to one of the most fascinating processes of biology, clarifying key questions such as whether cell-type-specific epigenetic marks are established during lineage priming prior to cell-fate commitment.

In addition, these experiments will have important applications in the clinic. For example, such information will assist efforts to reprogram cells from adult tissues into induced pluripotent stem cells (iPSCs). The inefficiency of this process is currently limiting the applicability of iPSCs to regenerative medicine, so single-cell gene expression analyses have been performed to decipher the molecular pathway to successful reprogramming [56–58]. Somatic cell reprogramming is known to be associated with dramatic nuclear remodeling [59, 60], so single-cell epigenomic studies will add an important layer of information. Furthermore, a detailed understanding of the mechanisms involved in cell-fate decisions in vivo will improve our ability to generate specific cell types (from iPSCs or other stem cells) for therapeutic use in regenerative medicine.

### Single-cell approaches to assess the complexity of cancer

Cancer is a highly heterogeneous disease with molecular characteristics that depend on the tissue of origin and differ between patients. Intratumor heterogeneity (within patients) is not fully understood, but includes regional differences that reflect tumor microenvironment, differences between primary and metastatic disease, and genetic diversity resulting from tumor evolution. Emerging single-cell sequencing technologies will reveal the full extent of intratumor heterogeneity and this will have many applications for clinical management as different cell types are likely to play distinct roles in disease initiation, metastasis, and drug resistance [61]. Already, single-cell DNA sequencing has found evidence of clonal evolution in multiple cancer types, and has identified founder mutations and subclonal mutations that have implications for cancer progression [62, 63]. Likewise, single-cell transcriptome profiling has been used to identify cell subpopulations within cancers, including cells with transcriptional programs suggesting stem cell activity [64–66]. These studies have extended our understanding of disease progression and have improved our ability to predict disease outcome.

The epigenome is known to be drastically remodeled in multiple malignancies, and therapeutics targeting DNA methyltransferases and histone deacetylases are used in several cancer types [67]. Typically, loss of DNA methylation is observed on a global scale while gains in DNA methylation occur in a more specific manner, and these changes are accompanied by abnormal nucleosome positioning and chromatin modifications. Descriptions of intertumor epigenetic heterogeneity have yielded clinically relevant information (e.g., stratification of triple-negative breast cancers into subgroups with differing prognosis [68]), but the full extent of epigenetic intratumor heterogeneity remains unknown and will rely upon single-cell analyses.

In the future, single-cell epigenomic studies will complement single-cell transcriptome and genome analysis in defining rare subpopulations of cells with clinically significant characteristics. For example, cancer stem cells could be characterized using these single-cell studies, such that targeted therapeutics can be designed to prevent disease recurrence following conventional therapy [69]. Single-cell epigenomic studies may also lead to the development of novel screening strategies based on circulating tumor cells and cell-free DNA, where patient material is severely limited. In particular, DNA methylation is an attractive target for cancer screening as it provides cell-type-specific information that is more stable than transcriptional profiles.

## Conclusions

**Fig. 2:**
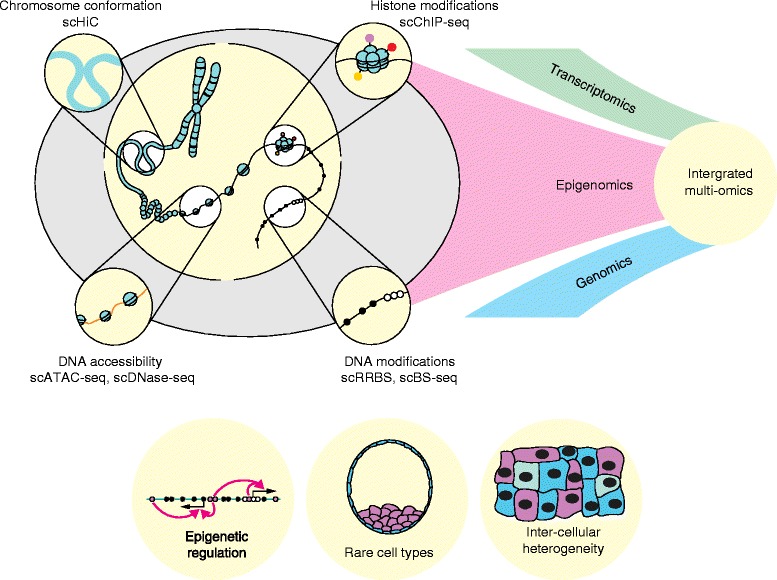
Future applications of single-cell epigenomics. The full potential of emerging single-cell epigenomic techniques will be realized through integration with transcriptome and genome sequencing. Single-cell multi-omics will be applied to biological questions involving the molecular mechanisms of epigenetic regulation (e.g., the functional consequences of rare DNA modifications), intercellular heterogeneity, and rare cell types (e.g., in early development). *scATAC-seq* single cell assay for transposase-accessible chromatin, *scBS-seq* single-cell bisulfite sequencing, *scChIP-seq* single-cell chromatin immunoprecipitation followed by sequencing, *scDNase-seq* single-cell DNase sequencing, *scHiC* single-cell HiC, *scRRBS* single-cell reduced representation bisulfite sequencing

In conclusion, the field of single-cell epigenomics is in its infancy but with the rapid pace of technological development and the increasingly recognized importance of intercellular heterogeneity we anticipate enormous progress over the next few years. Methods are evolving such that researchers will soon be able to profile multiple epigenetic marks within the same single cell and do so in combination with transcriptional and genetic information (Fig. 2). Correlations between features at precise genomic locations will lead to a more refined appreciation of how epigenetic processes interact with one another to control gene expression. Ultimately this has the potential to transform our understanding of how the phenotype of the cell is maintained and how it is perturbed in disease—a subject that is fundamental to biology.

## Abbreviations

5hmC: hydroxymethyl cytosine
5mC: methyl cytosine
ATAC-seq: assay for transposase-accessible chromatin
BS-seq: bisulfite conversion followed by sequencing
ChIP-seq: chromatin immunoprecipitation followed by sequencing
Dam: deoxyadenosine methylase
ESC: embryonic stem cell
FACS: fluorescence-activated cell sorting
iPSC: induced pluripotent stem cell
MNase: micrococcal nuclease
NOMe-seq: nucleosome occupancy and methylome sequencing
PCR: polymerase chain reaction
scBS-seq: single-cell BS-seq
scM&T-seq: single-cell methylome and transcriptome sequencing
scRBBS: single-cell reduced representation bisulfite sequencing
TAB-seq: TET-assisted bisulfite sequencing
